# Implementation of High-Flow Oxygen Therapy in a Surgical High-Dependency Unit: A Cohort Study

**DOI:** 10.7759/cureus.84857

**Published:** 2025-05-26

**Authors:** Joanne Chong Hui Ling, Bridget Ng Si Min, Shi Min Tan, Wei Qin Teo, Irene Too Ai Ling, Jolin Wong, Vui K Ho, Shin Yi Ng, Aarthi Suhitharan, Suhitharan Thangavelautham

**Affiliations:** 1 Specialty Nursing, Nursing Division, Surgical Intensive Care Unit, Singapore General Hospital, Singapore, SGP; 2 Surgical Intensive Care Unit, Department of Anesthesiology and Perioperative Sciences, Singapore General Hospital, Singapore, SGP; 3 Critical Care, Nursing Division, Surgical Intensive Care Unit, Singapore General Hospital, Singapore, SGP; 4 Anesthesiology, National Junior College, Singapore, SGP

**Keywords:** high-flow oxygen therapy, icu resource optimization, implementation, respiratory support, staff training, surgical high dependency unit

## Abstract

Background: High-flow oxygen therapy (HFOT) is a non-invasive respiratory support method traditionally used in the intensive care unit (ICU) settings for patients with acute hypoxemic respiratory failure. It delivers a consistent flow of humidified oxygen at high flow rates, improving oxygenation and reducing the work of breathing. The advantages of HFOT, such as its ability to provide a high fraction of inspired oxygen (FiO2) and its ease of use, have prompted its use beyond the ICU walls in various medical settings. This study examines the feasibility and safety of implementing HFOT in a surgical high-dependency unit (SHDU) at Singapore General Hospital (SGH), where it was introduced as part of a protocol to optimize patient care and ICU resource utilization.

Objective: The primary aim of this study was to evaluate the implementation of HFOT outside the ICU in SHDUs, assessing patient outcomes and the effectiveness of a structured training protocol for healthcare providers.

Methods: This cohort study was conducted in the SHDUs of SGH, a tertiary healthcare institution. The study population consisted of 89 patients who received HFOT across 96 administrations. A standardized HFOT protocol was developed to guide patient selection, initiation, monitoring, and management, with close supervision by the Rapid Response Team (RRT). A comprehensive staff training program was implemented, which included face-to-face training, online in-service education, and ongoing support for nurses. HFOT was delivered using the AIRVO™ 2 machine, which can provide up to 60 liters per minute of flow and an FiO2 of up to 0.95.

Results: A total of 96 HFOT administrations were analyzed, with the mean patient age being 70.8 years and 65% of patients being male. The average duration of HFOT was 40.2 hours. Of the 81 patients analyzed, 64 (66.7%) were successfully weaned to conventional oxygen therapy, while 24 (25%) required ICU-level ventilatory support. The implementation process involved a pilot phase in two SHDUs, followed by full-scale deployment across all units. However, the COVID-19 pandemic disrupted the process, leading to a temporary suspension of HFOT use in non-isolation wards for several months. Despite this setback, when restrictions were lifted, HFOT use outside the ICU expanded significantly, with a notable increase in the number of HFOT initiations in non-pilot wards. The implementation of HFOT outside the ICU faced challenges such as limited hands-on experience and logistical issues, which were addressed through structured training, additional equipment, and a mobile application for setup. Despite these efforts, delays and the lack of machine portability remained barriers to optimal implementation.

Conclusion: The successful implementation of HFOT in SHDUs at SGH highlights its feasibility as an effective alternative to ICU care for selected patients. More than two-thirds of patients who received HFOT were successfully managed without requiring escalation to ICU-level care. This study underscores the importance of structured protocols, staff education, and appropriate resource allocation in ensuring the safe and effective use of HFOT outside of the ICU.

## Introduction

High-flow oxygen therapy (HFOT) is a fixed-performance oxygen delivery device that provides a consistent concentration of warm, humidified oxygen at high flows [1-3]. It is a non-invasive form of advanced respiratory support conventionally used in the intensive care unit (ICU) for patients with acute type one (hypoxemic) respiratory failure [3-5]. It can be used as a nasal cannula or connected to a tracheostomy with an adaptor. Other uses of HFOT include pre-intubation oxygenation, where it serves as a bridge between mechanical ventilation and simpler forms of oxygen therapy post-extubation, treatment for acute pulmonary edema, and for reducing breathing work in patients with a do-not-resuscitate order [1,4,6-7]. The landmark randomized controlled trial on HFOT, the FLORALI study, demonstrated a significant reduction in intubation rate, ICU mortality, and 90-day mortality in patients treated with HFOT compared to those on conventional oxygen therapy [2].

The advantages of HFOT are that it can provide up to a fraction of inspired oxygen (FiO2) of 0.95 and a flow rate of up to 60 liters per minute, making it an efficient method of delivering high-flow oxygen to treat hypoxemic respiratory failure [3,5,8-9]. The high flows generate a modest effect of positive end-expiratory pressure (PEEP) at the alveolar level and effectively flush out dead space within the respiratory system [5-6,10]. The warm and humidified oxygen also maximizes comfort for the patient by reducing the nasal mucosal irritation that occurs with the administration of dry oxygen [5-6,8]. Furthermore, the administration of HFOT does not require the special skills or monitoring needed for non-invasive ventilation. Its ease of use makes it a feasible option in non-ICU settings [3].

However, in most hospitals, the use of HFOT is restricted to the ICU settings. One drawback associated with this is the potential delay in treatment for patients who require HFOT when there is no ICU bed available [10,11]. It also results in lower-acuity patients having to be moved to or to remain in the ICU to receive the therapy, generating healthcare costs and potentially denying a high-acuity patient ICU care [11]. 

Inadequate ICU bed capacity has been a major healthcare issue in many countries, and the COVID-19 pandemic emphasized the importance of the optimal use of ICU beds [12-13]. In recent years, there has been an increased popularity in the usage of HFOT outside of the ICU settings [5-6,11,14-15]. There have been reports on the use of HFOT beyond ICU walls, such as in general paediatric wards, emergency departments, trauma units, and mixed medical wards, but there is little evidence on the safety and efficacy of HFOT in adult surgical wards outside the ICU [5-6,11,14]. Expanding HFOT beyond the walls of the ICU is a challenge for any health institute when it involves nursing staff who have previously not looked after patients on HFOT [11]. A structured implementation of HFOT in non-ICU settings requires a meticulous protocol for appropriate patient selection and a well-planned staff education program to ensure staff comfort and acceptance [11]. Achieving patient safety and efficient use of HFOT relies heavily on successful implementation and staff buy-in [11]. This study examines the safety, efficacy, and feasibility of implementing HFOT outside of the ICU setting in a tertiary healthcare institution. 

## Materials and methods

Study design

This study examines the safety and feasibility of implementing HFOT outside of the ICU setting at Singapore General Hospital (SGH), a tertiary healthcare institution. At SGH, the surgical high-dependency units (SHDUs) provide invasive hemodynamic monitoring as well as cardiovascular and respiratory support to patients from all surgical disciplines. Patient care in SHDUs encompasses the administration of inotropic agents such as dopamine, supplemental oxygen therapy, and continuous monitoring by surgical teams. Additionally, critical care support is provided by the rapid response team (RRT) to ensure timely intervention and optimal patient management. A distinguishing feature of our institution is the dedicated SHDU for each surgical discipline. For instance, the general surgery (GS) department operates 20 SHDU beds distributed across two wards, while the orthopedics department manages 10 SHDU beds across two wards. This structure allows for specialized, discipline-specific perioperative care tailored to the needs of each patient population. The limitations of the SHDUs in our institution include ventilatory support (HFOT, non-invasive ventilation), continuous kidney replacement therapy, and the availability of respiratory therapists. HFOT was first introduced in the SGH surgical intensive care unit (SICU) in 2013. We piloted the use of HFOT in the GS SHDU in February 2019 and subsequently in the rest of the SHDUs months later. The AIRVO™ 2 by Fisher & Paykel was the primary machine used to deliver HFOT. This portable machine does not require a compressed air supply and can deliver a flow rate of up to 60 L/min and FiO2 of up to 0.95. 

The two main objectives of this study are the assessment of clinical outcomes to evaluate the safety and efficacy of HFOT implementation in SHDUs and to describe the operational challenges, such as staff education, availability of equipment, and unforeseen limitations due to the COVID-19 pandemic. 

Implementation strategy

After being implemented in 2018, the RRT identified the need to introduce the HFOT service as a part of the RRT to SHDUs and formed a multidisciplinary team, which included representatives from the RRT, Division of Surgery, and SHDUs nurses. Our strategy of implementation consisted of four steps. The implementation process began with the development of a standardized HFOT protocol to guide patient selection and ensure close monitoring upon initiation, prioritizing patient safety. The next phase involved a structured staff training program to equip healthcare providers with the necessary knowledge and skills. This was followed by a pilot implementation in two SHDUs to assess feasibility, conducted until sufficient HFOT machines were available and staff training was completed. The final phase involved the full-scale implementation of HFOT across all SHDUs, with the pilot phase specifically conducted in general surgical HDUs.

HFOT Protocol for SHDUs 

**Figure 1:**
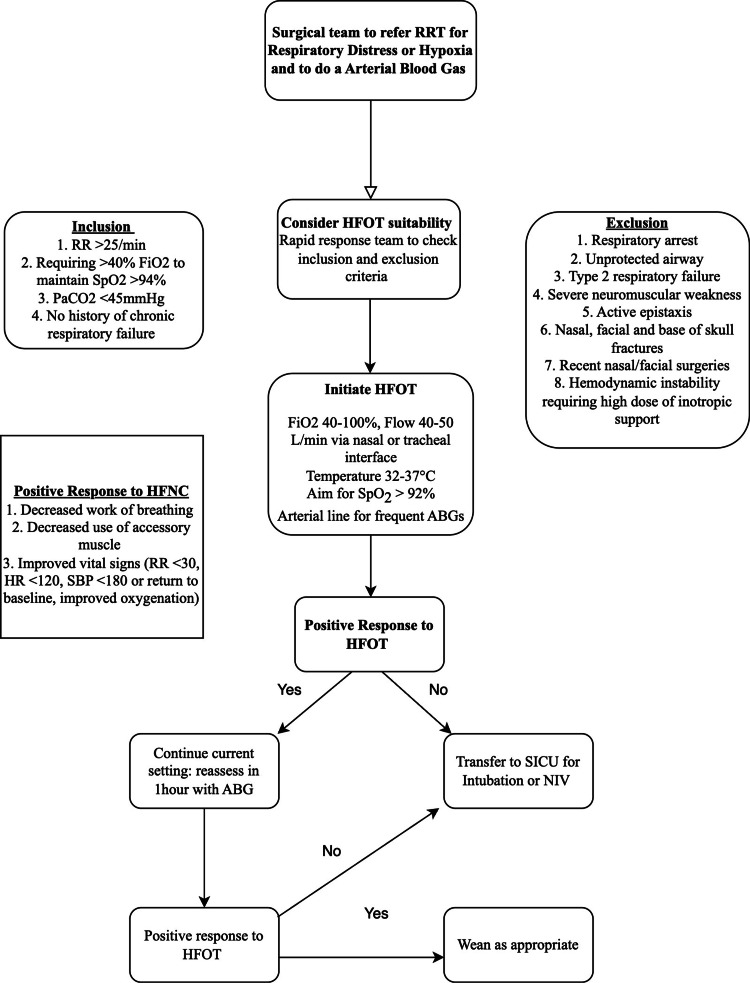
High-flow oxygen therapy protocol for surgical high-dependency units HFOT: high-flow oxygen therapy; HR: heart rate; NIV: non-invasive ventilation; PaCO2: partial pressure of carbon dioxide in arterial blood; RR: respiratory rate; RRT: rapid response team; SBP: systolic blood pressure; SICU: surgical intensive care unit

The implementation process commenced with the establishment of a standardized HFOT protocol to facilitate appropriate patient selection and ensure continuous monitoring at the start of therapy, with a strong emphasis on patient safety. When the surgical teams referred a patient to RRT for acute respiratory distress or hypoxia, the patients were assessed for the suitability of HFNC. Patients with respiratory distress were defined as having a respiratory rate > 25 breaths/min and requiring FiO2 >40% to maintain SpO2 > 94% with no history of chronic respiratory failure and were initiated on HFNC. This guideline was presented to the involved intensivists and the surgeons managing SHDUs and updated based on consensus. This protocol describes the workflow for identification of respiratory distress, inclusion and exclusion criteria for initiation of HFOT, monitoring after initiation and identification of positive response to HFOT, weaning protocol when the response is positive, and the escalation plan for those who do not show a positive response (Figure 1). 

The workflow facilitates early identification of high-risk patients, early detection of HFOT failure, and the need for intubation. To ensure the safety and effectiveness of treatment, only senior residents or intensivists from the RRT service were given the initiation and prescription rights. The patient had to be closely monitored in the SHDUs under the supervision of the RRT. An arterial blood gas test was done post-initiation to ensure a positive response, detect therapy failure, and prevent complications. Documentation was kept regarding the timing of initiation, machine settings, and the patient's vital signs. Patients on HFOT were monitored hourly, and doctors were to be alerted immediately if there was no improvement within the first hour of therapy to avoid delays in intubation. Nurses were also to inform doctors immediately if the patient's condition worsened, which was defined as exhibiting persistent or worsening respiratory status, experiencing hemodynamic instability, or deteriorating neurological status, as these are indicative of HFOT failure. The guideline outlined a weaning protocol, which only applied to nurses who were critical care trained and had been certified to titrate and wean patients off HFOT. We used AIRVO™ 2 (Fisher & Paykel, Auckland, New Zealand) for HFOT therapy. 

Training Protocol for HFOT Implementation

The subsequent phase entailed a comprehensive staff training program designed to provide healthcare providers with the essential knowledge and skills. Successful implementation of HFOT outside the ICU required comprehensive staff education and acceptance. The next phase involved a pilot implementation in two SHDUs to evaluate feasibility, continuing until an adequate number of HFOT machines were available and staff training was finalized. The final phase included the full-scale rollout of HFOT across all SHDUs, with the pilot phase specifically carried out in general surgical HDUs. The primary focus of the training was to equip nurses with the fundamental knowledge of HFOT, including its setup, application, and patient care considerations. A summary of the training protocol is outlined below, which includes face-to-face training sessions, online nursing in-service training, and ongoing patient monitoring follow-up.

Face-to-face training sessions: To ensure a thorough understanding of HFOT, all participating nurses attended face-to-face training sessions. These sessions covered key differences between traditional oxygen therapy and HFOT, patient selection criteria, contraindications, and the physiological and mechanical principles of the HFOT device. The training was divided into a theoretical component, delivered through presentations by the RRT nurses, and a practical hands-on session facilitated by vendors. To maximize engagement and encourage active participation, each session was limited to groups of 10 nurses. Competency assessment was conducted in a simulated environment where nurses practiced gathering relevant patient information, setting up the HFOT system, titrating oxygen delivery, troubleshooting issues, and disinfecting the equipment. This hands-on experience ensured that all participants were proficient in using the HFOT device before applying it in a clinical setting.

Online nursing in-service training: To maintain consistent and effective care for patients requiring HFOT in SHDU settings, an e-learning platform was implemented. This platform featured a PowerPoint presentation covering the theoretical aspects of HFOT, equipment setup, maintenance, and patient management. Additionally, a 10-question quiz was included as part of the continuing education program to reinforce key concepts. To further assist SHDU nurses in operating the AIRVO™ 2 device, instructional video links were provided. These videos offered guidance on the introduction, setup, and troubleshooting of the system, allowing nurses to refer to them whenever they encountered difficulties. This online training component complemented the face-to-face sessions, ensuring that nurses had continuous access to educational resources.

Follow-up and patient monitoring: To ensure patient safety and the effective application of HFOT, the RRT conducted twice-daily rounds in the SHDU. During these rounds, nurses documented key details in the electronic medical record, including the initiation time of HFOT, machine settings, and the patient's vital signs. Continuous monitoring was emphasized, with vital signs recorded hourly and arterial blood gas analysis performed one hour after HFOT initiation or earlier if the patient’s condition deteriorated. Routine skin assessments were conducted each shift to check for signs of pressure injuries, particularly in areas where the HFOT device made contact with the patient. To prevent skin breakdown, a protective hydrocolloid dressing was applied as needed. Additionally, oral hygiene and suctioning were performed at least once per shift to reduce the risk of infections. Infection control measures were strictly followed, with all consumables replaced according to manufacturer guidelines. If HFOT therapy was discontinued for more than 24 hours, all accessories were discarded to maintain hygiene and prevent contamination.

This structured training and follow-up protocol ensured that SHDU nurses were well-equipped to provide safe and effective HFOT care, ultimately improving patient outcomes in non-ICU settings.

Data collection

The following patient demographics and clinical characteristics were collected for analysis: age, gender, surgical discipline, type of surgery, and the underlying cause of respiratory failure. These variables provided a comprehensive overview of the patient population receiving HFOT in SHDU. Additionally, clinical outcomes were documented, with a particular focus on the incidence of successful HFOT weaning within the SHDU. This included assessing the proportion of patients who were able to transition off HFOT without requiring escalation to invasive mechanical ventilation or transfer to the ICU. This study was deemed exempt from IRB review by the SingHealth ethics committee, as it involved analysis of existing, de-identified data. 

Statistical analysis 

All categorical variables were presented as absolute counts and corresponding percentages, while continuous variables were summarized using mean values with standard deviations where applicable. The outcomes of HFOT therapy in the SHDU were calculated, and the results were expressed as percentages. 

## Results

Staff education

**Figure 2:**
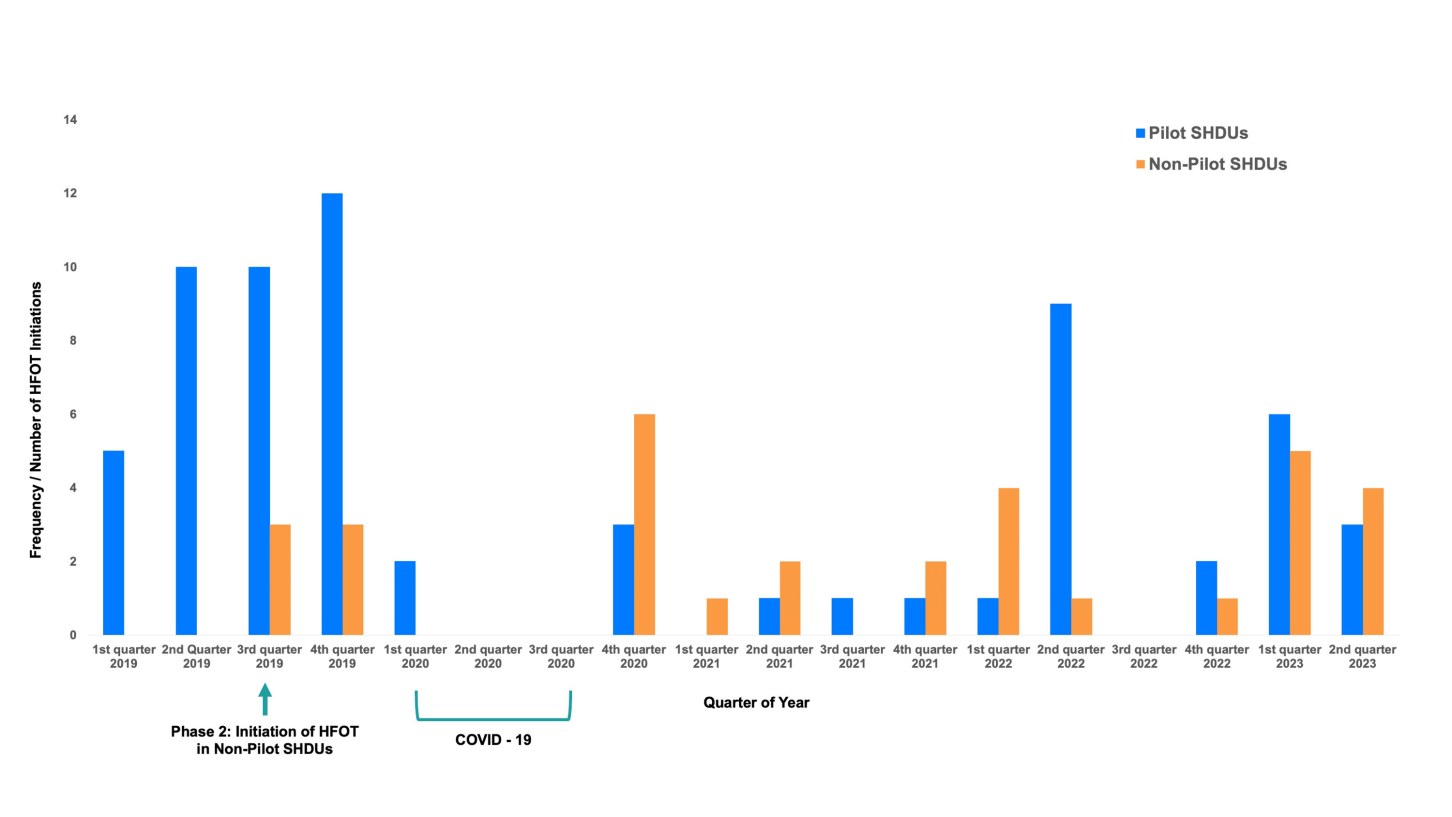
Numbers of high-flow nasal cannula initiations per quarter in SHDUs HFOT: high-flow oxygen therapy; SHDUs: surgical high-dependency units; blue: initiations in the pilot SHDUs; orange: initiations in non-pilot SHDUs; bracket: period of COVID-19 restrictions limiting the use of HFOT in SHDUs

A total of 53 SHDU nurses underwent onsite training for HFOT, but unfortunately, most of them were unable to gain practical experience with managing patients on HFOT due to COVID-19 restrictions in non-isolation wards. As a result of the restrictions, the use of HFOT outside of the ICU settings was suspended for up to six months, as shown in Figure 2.

Reinstatement of HFOT nursing training and implementation

During the COVID-19 period, the SHDU nurses provided verbal feedback about their lack of familiarity with HFOT due to infection control restrictions. To address this, the RRT reinforced the availability of video and refresher sessions for the nurses when patients require HFOT once the restrictions were eased. In addition, a video link on HFOT set-up and maintenance was provided to them for revision. As soon as the COVID-19 restrictions were lifted, an online nursing in-service course was implemented for all the 939 nurses, with 760 (81%) of them completing the online competency. 

Outcomes of HFOT implementation

Data were collected from 96 HFOT administrations across 89 patients in the SHDU. Outcome analysis was performed on 81 patients, including seven who had multiple HFOT initiations. Table 1 summarizes the characteristics of the 89 patients and the reasons for the 96 HFOT initiations. The mean age of the cohort was 70.8 years, with 58 patients (65%) being male. The average duration of HFOT treatment was 40.2 hours. 

**Table 1:** Demographics and clinical characteristics of patients before HFOT initiation HFOT: high-flow oxygen therapy; AMI: acute myocardial infarction; APO: acute pulmonary edema; COPD: chronic obstructive pulmonary disease; HAP: hospital-acquired pneumonia standard deviation = SD, SD expressed in the format means ±SD

Baseline characteristics	Total N = 89 patients, 96 HFOT initiations
Age, mean	70.8 ± 12.6
Length of admission (days), mean	32.4 ± 22.6
Gender	
Male	58 (65.2%)
Female	31 (34.8%)
Surgical discipline	
General surgery	55 (61.8%)
Vascular	10 (11.2%)
Orthopedics	11 (12.4%)
Urology	1 (1.1%)
Plastic surgery	1 (1.1%)
Obstetrics & gynecology	1 (1.1%)
Others	10 (11.2%)
Indications for initiation of HFOT	
Type 1 respiratory failure	86 (89.6%)
Pneumonia (HAP/aspiration)	28 (29.2%)
Fluid overload/APO/AMI	8 (8.3%)
Diaphragmatic splinting or intra-abdominal sepsis	6 (6.3%)
Multifactorial	26 (27.1%)
Non-specified	18 (18.8%)
Type 2 respiratory failure	8 (8.3%)
Pneumonia	2 (2.1%)
Fluid overload	1 (1.0%)
Multifactorial	2 (2.1%)
Non-specified	3 (3.1%)
Mixed respiratory failure	2 (2.1%)
Pneumonia (HAP/aspiration)	1 (1.0%)
Multifactorial	1 (1.0%)

Table 2 outlines the outcomes for patients initiated on HFOT. Of these, 64 patients (66.7%) were successfully weaned to standard oxygen therapy, while 24 patients (25%) required ventilatory support in the ICU.

**Table 2:** Outcome of HFOT in SHDU HFOT: high-flow oxygen therapy; SHDU: surgical high-dependency unit

Total numbers	Total N = 89 patients, 96 HFOT initiations
Outcome	
Wean to regular O2 device, n/total initiations (%)	64/96 (66.7%)
To intensive care unit, n/total initiations (%)	24/96 (25.0%)
To operating theater, n/total initiations (%)	2/96 (2.1%)
Withdrawal of HFOT for comfort care, n/total initiations (%)	6/96 (6.3%)

During the pilot phase of implementation in the first two quarters of 2019, two HFOT machines were used in the pilot wards, with a total of 15 HFOT initiations. Subsequently, in the third and final quarter of 2019, the use of HFOT outside ICU settings was extended to the rest of the SHDUs. This brought the total number of HFOT initiations outside ICU settings to 43, out of which six were in non-pilot wards (Figure 2). Due to COVID-19 restrictions in the hospital, the use of HFOT outside of ICU settings was temporarily halted during the first three quarters of 2020. However, in the fourth quarter of 2020, the use of HFOT was resumed. With the addition of more HFOT machines following the pandemic, a total of 26 out of 53 HFOT initiations were performed in the non-pilot SHDUs. This is a significant increase in the use of HFOT outside of the ICU since the COVID-19 pandemic, which potentially benefited many patients who require HFOT without needing ICU admission. 

## Discussion

This paper is the first to describe the role of proper patient selection, tight protocol, and nurse buy-in in the successful implementation of HFOT in surgical populations outside the ICU. More than two-thirds of patients were able to complete the treatment without requiring admission to the ICU. This is comparable [2] or higher [16] than the reported success rate of HFOT therapy in ICU patients. This highlights the potential for HFOT to be effectively administered outside the ICU, reducing ICU bed strain and workload [17]. 

HFOT involves patients with acute hypoxic respiratory failure who are at risk of rapid deterioration into severe hypoxia or multiorgan failure. Starting HFOT inappropriately or without close monitoring in a non-ICU environment can indeed lead to delayed intubation, which is associated with poor outcomes [15]. This is particularly relevant in cases such as aspiration of gastric contents, where immediate intubation might be more appropriate [18]. A stringent protocol was developed to regulate the initiation of HFOT, stipulating that only critical care-trained personnel are authorized to initiate therapy. The protocol also ensures close monitoring following initiation and includes specific criteria to identify HFOT failure. Our findings demonstrate the safety and efficacy of implementing HFOT outside the ICUs, with results comparable to those reported by Jackson et al. in 2021 [11].

The nurses' proficiency and competency in setting up and managing the HFOT equipment were a challenge in the implementation of this therapy [19]. Since the use of HFOT was infrequent at the beginning, nurses in the SHDU had limited opportunities to operate the equipment or manage patients being treated with HFOT. As a result, the lack of hands-on practice made it difficult for the nurses to recall the necessary steps for setting up the equipment, even though they had received prior training. This often led to situations where they had to ask for assistance from ICU nurses. Furthermore, the high turnover rate among nursing staff meant that new staff required training on the use of HFOT. 

To address these issues, the team collaborated with the nursing managers of the SHDU to identify a core group of nurses who would be responsible for conducting training on the use of HFOT for both experienced nurses who required revision and new hires. The RRT nurses introduced the AIRVO™ 2 mobile application to help SHDU nurses set up and use the HFOT equipment. The app provides a visual guide for the process. The team also monitored the patient's documentation in the electronic medical records while on HFOT to ensure accurate recording of critical values. Inconsistent or incomplete documentation by staff was addressed through re-education. These measures allowed nurses to become more familiar and comfortable with the use of HFOT.

Despite the overall success, the team has identified two barriers related to the use of HFOT. One problem is the limited mobility of patients using the AIRVO™ 2 HFOT machine, which does not come with a built-in battery. This means that patients cannot be transported to other departments for procedures or scans while using the machine. Also, patients who need to be mobilized for physiotherapy cannot use the HFOT, which forces them to switch to an alternative oxygen therapy, such as the venturi mask. However, this may cause the loss of PEEP in the alveoli, leading to increased breathing difficulty for the patients. It takes time to recruit the lungs again after restarting the HFOT, which may prolong the therapy. To address this issue, the team may consider installing a portable power source on the HFOT machines to allow for transport and mobilization.

The second issue was the limited availability of HFOT machines in every SHDU. During the initial phase of the implementation, two HFOT machines leased from the vendors were shared between three SHDUs. A few patients in SHDUs who had required HFOT experienced delays in the initiation of therapy while the staff acquired the machine and equipment from another unit. However, with the compelling preliminary results of the study, we were able to convince the hospital management to fund the procurement of five HFOT machines for the use in SHDUs.

Limitations 

It is important to acknowledge that there are specific limitations that need to be considered. Unfortunately, a few months after initiation, the pandemic started. Hence, the hospital had to impose limitations on the usage of HFOT due to COVID-19 restrictions and concerns regarding aerosol transmission for more than a year. These restrictions meant that HFOT could only be used in isolation wards, and there were strict criteria that needed to be met before initiating HFOT outside these wards. For example, patients were required to have two negative COVID-19 swabs before beginning HFOT outside isolation wards. Due to these limitations, some SHDU patients with acute deteriorating conditions had to be sent to SICU or isolation wards for HFOT instead, which was reflected in the observed graph trend. COVID-19 restrictions not only negatively influenced the recruitment rate at SHDUs but also led to attrition of recently acquired knowledge on HFOT management among the nurses. However, after all COVID-19 restrictions were lifted and the hospital purchased additional HFOT machines, which were distributed between SICU and several SHDUs, there was an increase in the trend of HFOT usage in non-pilot wards. This allowed for more HFOT to be initiated outside of the ICU settings.

## Conclusions

The implementation of HFOT in the SHDUs at our institution has demonstrated its feasibility and safety outside the ICU setting. By establishing a structured protocol, ensuring careful patient selection, and prioritizing comprehensive staff education, we successfully expanded HFOT use while maintaining patient safety. Our findings suggest that more than two-thirds of patients on HFOT were effectively managed in SHDUs without requiring ICU escalation, underscoring the potential of HFOT in optimizing ICU resource utilization. This indicates that careful patient selection can enable many patients to receive HFOT outside the ICU, while still achieving positive outcomes.

Despite initial challenges, including limited hands-on experience for nurses due to COVID-19 restrictions and logistical barriers such as equipment availability and patient mobility, our approach facilitated a gradual but impactful integration of HFOT in SHDUs. Addressing these challenges through ongoing training, enhanced documentation practices, and increased equipment allocation further strengthened the sustainability of the program. Moving forward, continued evaluation and refinement of the HFOT implementation strategy will be essential to maximize its benefits, ensuring that critically ill surgical patients receive timely and effective respiratory support without unnecessary ICU admissions. 
